# Detergent Screening With Hybrid Detergents Increases Observable Number of Protein Identities in Bottom‐Up Proteomics

**DOI:** 10.1002/pmic.70003

**Published:** 2025-06-20

**Authors:** Jan‐Simon Behnke, Andreas Hentschel, Maximilian Wolf, Virginia Wycisk, Albert Sickmann, Robert S. Heyer, Leonhard H. Urner

**Affiliations:** ^1^ Department of Chemistry and Chemical Biology TU Dortmund University Dortmund Germany; ^2^ Leibniz‐Institut für Analytische Wissenschaften—ISAS—e.V. Dortmund Germany; ^3^ Faculty of Technology Bielefeld University Bielefeld Germany

## Abstract

Detergents are key reagents in bottom‐up proteomics that create an apparent, yet underappreciated bias on observable proteomes. Maximizing the chemical diversity of detergents in parallelized screens is supposed to maximize observable proteomes if proteomics data sets of different detergents are combined. The aim of our work is to investigate the potential of fusing ionic and nonionic detergent headgroups into hybrid detergents for increasing the observable number of unique protein identities. Our data indicate that the solubilizing properties of hybrid detergents do not reflect an average of canonical detergents. The number of unique protein identities obtainable from an *Escherichia coli* screen increases from 1604 to 2169 when proteomics data sets from sodium dodecyl sulfate, dodecyltrimethylammonium bromide, dendritic triglycerol detergent, and related hybrid detergents are combined. Our data highlight the utility of cationic detergents and related hybrid detergents for enhancing observable proteomes. Detergent screening–based proteome reconstructions with canonical detergents and hybrid detergents present an interesting research direction towards improved proteome profiling applications.

## 1 Introduction

Proteomics aims for the identification of the entirety of all proteins within cells or organisms at a certain time point. Since biomolecular interactions translate into biological function, proteomics is exceptionally useful for the description of biological systems, including the search for disease‐relevant signaling cascades, biomarkers, monitoring of disease development and drug discovery [1, 2, 3, 4]. Technologies that enable proteomics are of great interest to life sciences and the pharmaceutical industry [5, 6].

To release proteomes from cellular environments for proteomics, detergents are commonly used [1, 4]. Detergents are amphiphilic molecules that can help with releasing proteomes by lysing cells and solubilizing proteins. The structure of a detergent creates a bias for obtainable protein identities (protein IDs). This bias isa determined empirically and aligned with experimental goals. Sodium dodecyl sulfate (SDS) is a widely used standard that gives large numbers of protein IDs in bottom‐up proteomics [1, 4]. Complementary, nonionic detergents, such as Triton X‐100, NP‐40, Brij, or bile acids, like, sodium deoxycholate, are commonly used [1, 4]. Interestingly, cationic detergents are underrepresented in proteomics. Choi and co‐workers proposed that combining proteomics data sets from different detergents, including cationic benzalkonium chloride, can increase the number of unique protein IDs observed in bottom‐up proteomics [7]. To stimulate knowledge gain in this direction, we established ionic/nonionic hybrid detergents by fusing ionic with nonionic detergent headgroups [8, 9]. Ionic/nonionic hybrid detergents resemble covalent combinations of ionic and nonionic detergent headgroups whose potential for bottom‐up proteomics remains to be explored. Herein, our aim is to investigate the question of whether ionic/nonionic hybrid detergents can increase observable numbers of unique protein IDs if proteome data sets from a parallelized detergent screening on *Escherichia coli (E. coli)* are combined.

## 2 Methods

### 2.1 Detergent Synthesis

**FIGURE 1:**
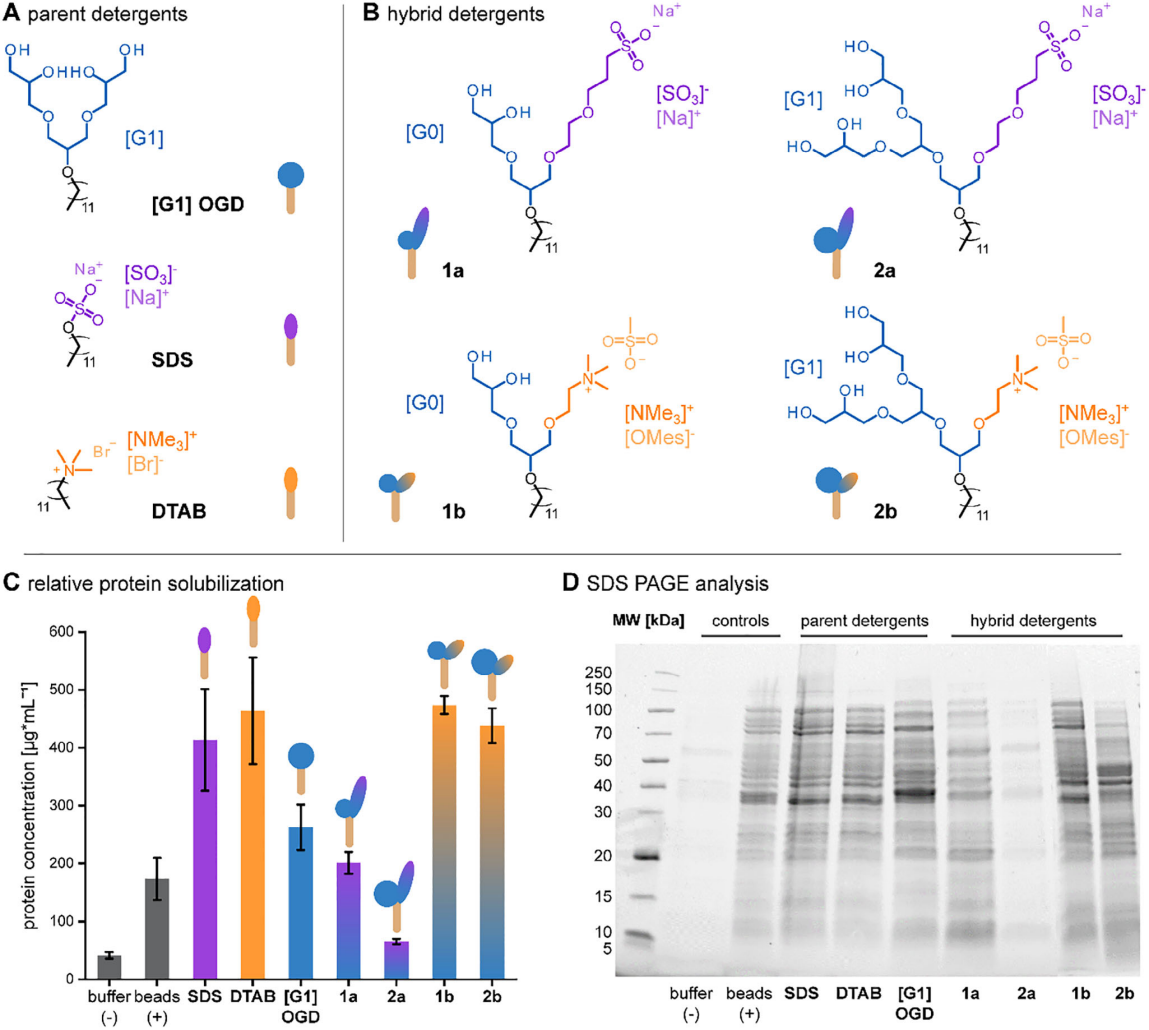
Utilized detergents for proteomics and solubilized *E. coli* proteomes. (A) Molecular structures of parent detergents (SDS, DTAB, [G1] OGD) and (B) structurally related ionic/nonionic hybrid detergents (**1a**, **2a**, **1b**, **2b**). (C) Bar chart showing relative protein concentrations solubilized by buffer (negative control), glass beads (mechanical control), and detergents under comparable conditions. (D) SDS PAGE gel showing solubilized *E. coli* proteomes against different detergents.

Anionic SDS (99%, Thermo Fischer) and cationic dodecyltrimethylammonium bromide (DTAB) (99%, Thermo Fischer) were used as supplied. The nonionic detergent [G1] OGD was synthesized in‐house as described before [10]. The ionic/nonionic hybrid detergents were also synthesized in‐house as described before [8, 9]. The chemical nature of polar headgroups determines protein solubilization and could principally be biased by other polar groups [1, 4]. To minimize the bias of detergent chemistry on proteomics data, we excluded atom groups in our hybrid detergents other than those present in parent detergents (Figure 1A,B) [11].

### 2.2 Cell Lysis and Sample Preparation

To generate sufficient *E. coli* cells for lysis, an *E. coli* K12 MG1655 [M. S. Guyer strain, purchased from the DSMZ (Germany), DSM No.: 18039] colony was picked from Luria‐Miller (LB) broth agar‐plates (4‐g LB‐Agar‐Mix, 100‐mL deionized water) and transferred into 20‐mL LB medium (2.5 g per 100‐mL deionized water). The mixture was incubated at 37°C with 180 rpm for 3.5 h. The bacterial suspension was diluted with sterile LB broth medium to an OD_600_ of 1.1. The supernatant was clarified by centrifugation (4°C, 4000 *g*, 10 min) and discarded. The remaining cell pellet was resuspended in 19 mL freshly prepared wash buffer (50‐mM Tris, pH = 7.5, 0.1‐mg/mL chloramphenicol, 1‐mM phenylmethylsulfonyl fluoride). The supernatant was clarified (4°C, 4000 *g*, 10 min) and discarded. The cell pellet was resuspended in 19 mL freshly prepared wash buffer and split into 1.4‐mL aliquots (1.5‐mL Eppendorf tubes, Protein LoBind). The supernatant was clarified (4°C, 13,000 *g*, 10 min) and discarded.

To lyse cells, every cell pellet was resuspended with 300‐µL lysis buffer (20‐mM Tris, pH = 7.5, 30‐mM detergent of interest). As a negative control, no detergent was added. As mechanical control, instead of detergent, 300‐mg glass beads (diameter 0.5 mm) were added [12]. The tubes were incubated at 60°C with 1500 rpm for 5 min followed by another incubation step at 37°C with 1500 rpm for 10 min [13]. The supernatants were clarified (4°C, 13,000 *g*, 10 min), and stored at 4°C for direct SDS polyacrylamide gel electrophoresis (PAGE) analysis or at −20°C for long‐term storage. Solubilized protein concentrations were determined with the Bicinchoninic acid (BCA) assay (Pierce BCA Protein Assay Kit, Thermo Scientific, USA) in a 96‐well plate format according to manufacturer instructions [14].

### 2.3 SDS PAGE Analysis

The presence of detergents in solubilized protein samples interfered with protein separation during SDS PAGE analysis (Figure S1). Therefore, detergents were removed by mixing a volume of clarified lysate containing 27.5 µg protein with 5× volume of cold acetone (−20°C) [15]. The samples were stored at −20°C overnight. The supernatant was clarified (4°C, 4000 *g*, 30 min), discarded and residual acetone was evaporated under a fume hood for 10 min at room temperature. So‐obtained precipitates were solubilized with deionized water (15 µL) and sample buffer (5 µL of ROTILoad 1 4×, Carl Roth, Germany) followed by incubation at 90°C for 5 min. The samples were analyzed by SDS PAGE using stain‐free gels (mini‐PROTEAN TGX Stain‐Free, Bio‐Rad, USA) in running buffer (25‐mM Tris, 192‐mM glycine, 0.1 wt% SDS) with a SDS PAGE station (voltage: 200 V, run duration: 30–45 min, Bio‐Rad). Gels were imaged in a ChemiDoc MP station (Bio‐Rad) with 5 min of light activation (Figure S2).

### 2.4 Sample Preparation for LC–MS/MS

For LC–MS/MS, the clarified supernatants that were prepared as described in the subsection **“**Cell lysis and sample preparation” were flash‐frozen in liquid nitrogen and shipped on dry ice from TU Dortmund University to the Leibniz Institute for Analytical Science. Samples were thawed and protein concentrations in the supernatants were determined using the BCA (Pierce BCA Protein Assay Kit, Thermo Scientific, USA) according to the manufacturer protocol. Subsequently, disulfide bonds were reduced by the addition of 10‐mM Tris‐(2‐carboxyethyl)‐phosphine at 37°C for 30 min, and free sulfhydryl bonds were alkylated with 15‐mM iodoacetamide at ambient temperature in the dark for 30 min. Following the user manual, 100‐µg protein of each sample was used for proteolysis using the S‐Trap protocol (ProtiFi) and a protein‐to‐trypsin ratio of 20:1. The incubation time for trypsin was 2 h and done at 37°C. Proteolysis was stopped using formic acid to acidify the sample (pH < 3.0).

All proteolytic digests were checked using a previously established procedure [16]. Digests were controlled using monolithic column separation (PepSwift monolithic PS‐DVB PL‐CAP200‐PM, Dionex) on an inert Ultimate 3000 HPLC (Dionex, Germany) by direct injection of 1‐µg sample. A binary gradient (solvent A: deionized water + 0.1% trifluoroacetic acid; solvent B: 84% acetonitrile + 0.08% trifluoroacetic acid) ranging from 5% to 12% B in 5 min and then from 12% to 50% B in 15 min at a flow rate of 2.2 µL/min and at 60°C was applied. UV traces were acquired at 214 nm and displayed in the Supporting Information to confirm favorable protein digest (Figures S3–S9). The absence of peaks between 17.50 and 21.25 min is diagnostic for favorable protein digests [16]. These spectra indicate no interference of detergents with protein digest; however, signal suppression effects cannot be fully excluded.

### 2.5 LC–MS/MS Measurements

A total of 1 µg of the respective, digested peptide sample was separated on an Ultimate 3000 Rapid Separation Liquid Chromatography (RSLC) nanosystem with a ProFlow flow control device coupled to a Q Exactive HF orbitrap mass spectrometer (Thermo Scientific, Germany). For peptide concentration, a trapping column was used (Acclaim C18 PepMap100, 100 µm, 2 cm, Thermo Fisher Scientific, Germany); water + 0.1% trifluoroacetic acid (Sigma‐Aldrich, Germany), and operated with a flowrate of 10 µL/min. To separate peptides over a reversed‐phase column (Acclaim C18 PepMap100, 75 µm, 50 cm (Thermo Fisher Scientific), we used a binary gradient (solvent A: deionized water + 0.1% formic acid (Sigma‐Aldrich, Germany)/solvent B: 84% acetonitrile + 0.1% formic acid (Sigma‐Aldrich, Germany); 5% solvent B for 3 min, linear increase to 25% solvent B for 102 min, a further linear increase to 33% solvent B for 10 min, and then a final linear increase to 95% solvent B for 2 min followed by a linear decrease to 5% B for 5 min. For MS survey scans, the following settings were used: MS was operated in data‐dependent acquisition mode (DDA) with full MS scans from 300 to 1600 *m*/*z* (resolution 60,000) with the polysiloxane ion at 371.10124 *m*/*z* as lock mass. Maximum injection time was set to 120 ms. The automatic gain control (AGC) was set to 1 × 10^6^. For fragmentation, the 15 most intense ions above the threshold ion count of 5 × 10^3^ were chosen at a normalized collision energy (nCE) of 27% in each cycle, following each survey scan. Fragment ions were acquired (resolution 15,000) with an AGC of 5 × 10^4^ and a maximum injection time of 50 ms. Dynamic exclusion was set to 15 s.

### 2.6 Data Analysis

**FIGURE 2:**
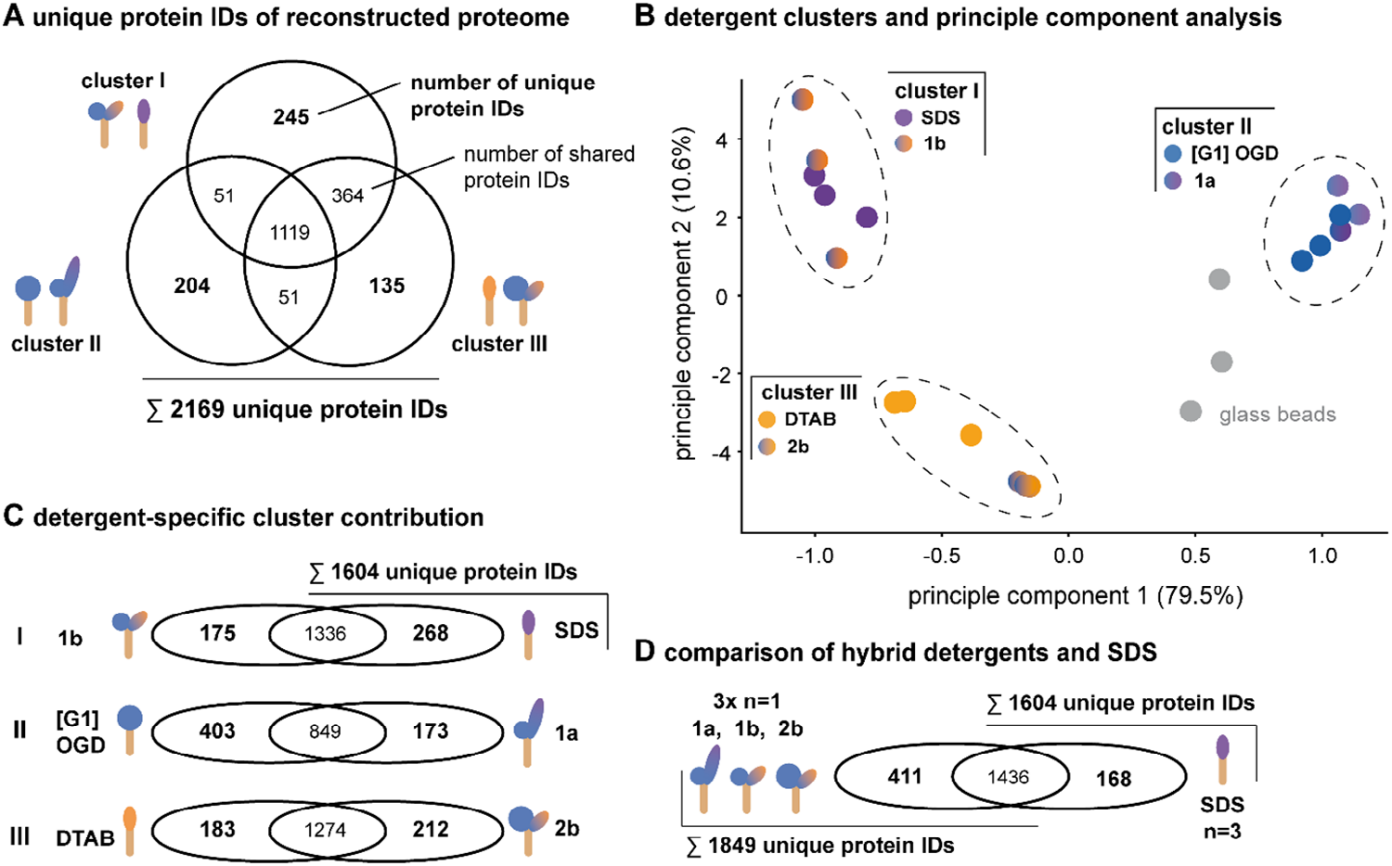
Reconstructed *E. coli* proteome from the detergent screen with hybrid detergents. (A) Venn diagram showing contributions of detergent clusters I, II, and III to the total number of unique protein IDs identified by the combined proteomics data set. (B) PCA of identified proteins by detergent. Color‐coded dots represent independent biological repeats (*n* = 3 per detergent). (C) Venn diagrams showing detergent‐specific contributions to unique protein IDs within the Clusters I, II, and III. (D) Venn diagram showing the number of unique protein IDs obtained from merged analysis of hybrid detergents **1a**, **1b**, and **2b** (3× *n* = 1) compared to SDS (*n* = 3) under comparable conditions.

For all data processing, the Proteome Discoverer software 2.5 (Thermo Scientific, Schwerte, Germany) was used and searches were done in a target/decoy mode against an *E. coli* UniProt database (UniProt) (www.uniprot.org) using the MASCOT and SEAQUEST algorithm. The following search parameters were used: precursor and fragment ion tolerances of 10 ppm and 0.02 Da for MS and MS/MS; a trypsin set as the enzyme with a maximum of two missed cleavages; carbamidomethylation of cysteine set as the fixed modification and the oxidation of methionine was set as a dynamic modification; and using a Percolator false discovery rate set to 0.01 for both peptide and protein identifications. A label‐free quantification (LFQ) analysis was performed for each condition. Proteins were considered as significantly regulated with *p* value of 0.05 after identification with at least two unique peptides and a ratio of 2 (two‐fold enrichment) or 0.5 (two‐fold downregulation). To estimate the cellular location of proteins, we utilized gene ontology analysis, where the UniProtKB entries were entered into the gene ontology webtool (www.geneontology.org) [17, 18]. To obtain the average isoelectric point (pI), the UniProtKB entries were entered into the expasy webtool “compute PI/MW” and the sum of pIs was divided by the number of entries [19] (Table 1). To obtain average molecular weights, the UniProtKB entries were entered into the expasy webtool “compute PI/MW” and the sum of molecular weights was divided by the number of entries (Table 1). To obtain a principal component analysis (PCA), the Python packages scipy (1.13.0) and scikit‐learn (1.4.2) were used to analyze proteomes solubilized by detergents and glass beads (Figure 2B).

**TABLE 1:** Parameters of *E. coli* proteomes identified by different detergents. Summary of parameters related to bottom‐up proteomics analysis from LC–MS/MS analysis of digested samples derived from the lysis of *E. coli* K12 MG1655 with beads and different detergents ([G1] OGD, SDS, **1a**, DTAB, **1b**, **2b**).

Parameters	Beads	SDS	[G1] OGD	1a	DTAB	1b	2b
Protein concentration [µg/mL]	174	414	263	201	464	474	438
Total identified peptides	12,328	16,688	12,229	9,774	15,111	16,232	14,873
Total identified proteins	1318	1604	1252	1022	1457	1511	1486
Identified membrane proteins via gene ontology analysis	317	395	284	255	321	367	331
% Membrane proteins via gene ontology analysis	24.1	24.6	22.7	25.0	22.0	24.3	22.3
Average MW [kDa]	38	38	35	34	36	38	37
Average pI	6	6	6	6	6	6	6

## 3 Results and Discussion

To assess whether fusing detergent headgroups into hybrid detergents can help to maximize the observable proteome in detergent screening–based proteome reconstruction, we designed a detergent library containing anionic SDS, cationic DTAB, nonionic [G1] OGD as well as structurally related ionic/nonionic hybrid detergents (**1a**, **2a**, **1b**, **2b**) (Figure 1A–B).

To assess how fusing ionic and nonionic detergent headgroups affects relative protein concentrations during lysis, we solubilized *E. coli* K12 MG1655 under comparable conditions with our detergents. We included solubilization trials with detergent‐free buffer as a negative lysis control and glass beads as a mechanical lysis control [12]. A commonly used concentration of SDS for lysis is 30 mmol [20], which we also used for other detergents to do a comparable structure‐property study. The utilized detergent concentrations were well above their critical micelle concentrations to secure the solubilization of hydrophobic cell components (Table S1) [21]. The solubilized protein concentrations were quantified with a BCA assay and plotted against the detergent abbreviations (Figure 1C) [14]. In the cases of parent detergents, solubilized protein concentrations were sensitive to the presence of a charged headgroup. Higher protein concentrations between 400 and 500 µg/mL were solubilized in the cases of anionic SDS and cationic DTAB (Table 1 and Figure 1C). Lower protein quantities between 200 and 300 µg/mL were obtained from nonionic [G1] OGD and the anionic/nonionic hybrid detergent **1a** (Table 1 and Figure 1C). Solubilized protein concentrations were not solely determined by charge. For example, cationic hybrid detergents **1b** and **2b** delivered similar protein quantities compared to DTAB and SDS, while anionic hybrid detergents delivered lower protein concentrations compared to DTAB and SDS (Table 1 and Figure 1C). This is expected because *E. coli* membranes exhibit a negatively charged outer membrane surface, which causes attractive electrostatic interactions with cationic detergents and improves their membrane‐damaging properties [9].

Noticeably, the anionic hybrid detergent **2a** led to similar protein concentrations as obtained for the negative control, that is, <80 µg/mL (Table 1 and Figure 1C). We conclude that the ability to solubilize *E. coli* with detergents depends on both the charge and size of the nonionic head of hybrid detergents. Fusing detergent headgroups into hybrid detergents does not result in an average of their solubilizing properties.

To investigate how fusing ionic and nonionic detergent headgroups affects the identity of solubilized proteins, we compared qualitatively the solubilized proteomes by SDS PAGE analysis (Figure 1D). To minimize the effect of detergents on image quality, we freed the solubilized protein samples from detergents by acetone precipitation and analyzed comparable protein amounts by SDS PAGE analysis [15]. Qualitatively, despite many similarities in band profiles, the SDS PAGE images obtained from all detergents were not identical (Figure 1D). Even though we are currently not able to rationalize how the detergent structures led to this experimental outcome, our data indicate that the solubilized proteomes vary with the chemical nature of the detergent headgroups (Figure 1D).

To investigate whether the obtained differences in solubilized protein concentrations and SDS PAGE band profiles reflect differences in solubilized proteomes, we analyzed all protein extracts by LC–MS/MS analysis following an on‐column trypsin digest and compared numbers of identified peptides and related proteins (Table 1) [22, 23]. We excluded the negative lysis control and the detergent **2a**, as their cell lysis yielded incomparable amounts of protein (Figure 1C).

In line with the relative solubilized protein concentrations obtained upon lysis, we identified more peptides in the cases of anionic SDS, cationic DTAB and cationic hybrid detergents **1b**, **2b** compared to [G1] OGD and **1a** (Table 1). We observed the same trend for the absolute number of identified proteins (Table 1). Even though variable numbers for total identified peptides and proteins varied between detergents, the gene ontology analysis obtained from the individual proteomes was in a narrow range of 22%–25% (Table 1). This indicates that all detergents solubilized comparable relative numbers of identified membrane‐associated proteins (Table 1).

To estimate the efficiency of our detergent screening–based proteome reconstruction with canonical detergent and hybrid detergents, we combined all observable proteomes which led to a total number of 2169 unique protein IDs (Figure 2A). The maximum number of unique protein IDs encoded in the genome of *E. coli* K12 is about 4285 [24]. Our findings suggest that detergent screening–based proteome reconstruction could improve the number of observable unique protein IDs, compared to SDS alone. However, this comparison is not fair, since SDS (*n* = 3) was compared with a merged analysis containing SDS and five detergents ([G1] OGD, **1a**, DTAB, **1b**, **2b**) (*n* = 18).

To evaluate whether hybrid detergents can be used to increase the observable proteomes compared to SDS using comparable sample sizes, we compared the combined proteomes of **1a**, **1b**, **2b** (*n* = 3) with SDS (*n* = 3) (Figure 2D). The merged analysis containing **1a**, **1b**, and **2b** again increased the number of unique protein IDs from 1604 to 1849 compared to SDS (Figure 2D).

To investigate the contributions of individual detergents in maximizing the number of observable unique protein IDs, we compared the similarity of individual proteomics data sets with a PCA (Figure 2B) [25, 26]. Following this method, the similarity between the proteomes obtained from two detergents increases the closer the distance between the related data points in our PCA. We identified three clusters of proteomes that share similar unique proteins, that is, cluster (I) SDS and **1b**, (II) [G1] OGD and **1a**, (III) DTAB and **2b** (Figure 2B). This confirms that different SDS PAGE profiles observed before reflect indeed different proteomes (Figure 1D).

Furthermore, parallel detergent screens with ionic/nonionic hybrid detergents, nonionic [G1] OGD, as well as cationic DTAB can help to complement the observable number of unique protein IDs that are observable with SDS (Figure 2A). All detergent clusters had about 1119 unique protein IDs in common (Figure 2A). Between 135 and 245, unique protein IDs were exclusively observed within the individual detergent clusters (Figure 2A). Interestingly, the detergent pairs within cluster (I) or (II) differed in terms of charge and size of the nonionic backbone. When it comes to proteomics, ionic/nonionic hybrid detergents represent no linear average of the solubilizing properties of individual detergents. In fact, hybrid detergents are unique structures and reflect unique properties [11, 27]. The same is true for different proteins. Since proteins can require individual solubilization conditions, combining data sets from detergent screening–based proteome reconstruction with hybrid detergents can help with maximizing the number of unique protein IDs in proteome reconstructions.

## 4 Conclusion

In summary, we established the utility of ionic/nonionic hybrid detergents for increasing the number of observable unique protein IDs from an *E. coli* proteome in a detergent screen format. Hybrid detergents and canonical detergents enabled the solubilization of *E. coli* proteins, SDS PAGE analysis, and LC–MS/MS analysis following trypsin digest. Merging the analysis of proteomics data sets from hybrid detergents presents a complementary approach to increase the number of observable unique protein IDs compared to SDS under comparable conditions and sample sizes (Figure 2D). Future studies will need to clarify the utility of hybrid detergents in enriching cell organelles and compatibility with mass spectrometry, including a detergent‐specific characterization of optimal solubilization conditions. Given that the cellular environment of interest is available in sufficient quantities, detergent screening–based proteome reconstructions with canonical detergents and hybrid detergents will improve the number of observable protein IDs in future proteome profiling applications.

## Author Contributions

R.S.H. and L.H.U. conceptualized the project. L.H.U., J.‐S.B., and V.W. designed, synthesized, and characterized [G1] OGD and hybrid detergents. J.‐S.B., A.H., M.W., and A.S. performed proteomics experiments and data analysis. The manuscript was written by L.H.U. with input from all authors.

## Conflicts of Interest

The authors declare no conflicts of interest.

## Supporting information




**Supporting information file 1**: pmic70003‐sup‐0001‐SuppMat.docx

## Data Availability

The mass spectrometry proteomics data have been deposited to the ProteomeXchange Consortium via the PRIDE [28] partner repository with the dataset identifier PXD060875.
