# Supplementary material for: Detergent Screening With Hybrid Detergents Increases Observable Number of Protein Identities in Bottom‐Up Proteomics
Source: Proteomics. 2025 Jun 20;25(15):6–12. doi: 10.1002/pmic.70003 (PMC12329390; doi:10.1002/pmic.70003)
Supplement: Supplementary file 1 — Supporting information file 1: pmic70003‐sup‐0001‐SuppMat.docx [file PMIC-25--s001.docx]

Supporting Information

**Detergent Screening with Hybrid Detergents Increases Observable Number of Protein Identities in Bottom-Up Proteomics**

Jan-Simon Behnke,^a^ Dr. Andreas Hentschel,^b^ Maximilian Wolf,^c^ Virginia Wycisk,^a^ Prof. Dr. Albert Sickmann,^b*^ Prof. Dr. Robert S. Heyer,^c,d*^ Dr. Leonhard H. Urner^a*^

a TU Dortmund University, Department of Chemistry and Chemical Biology, Otto-Hahn-Str. 6, 44227 Dortmund, Germany

b Leibniz-Institut für Analytische Wissenschaften – ISAS – e.V., Otto-Hahn-Str. 6b, 44227 Dortmund, Germany

c Bielefeld University, Faculty of Technology, Universtitätsstraße 25, 33615, Bielefeld, Germany

d Leibniz-Institut für Analytische Wissenschaften – ISAS – e.V., Bunsen-Kirchhoff-Str. 11, 44139 Dortmund, Germany

* corresponding authors: albert.sickmann@isas.de, robert.heyer@isas.de, leonhard.urner@tu-dortmund.de

Table of Contents

[1. Supplementary Figures 1](#_Toc196827624)

[2. Supplementary Tables 9](#_Toc196827625)

# 1. Supplementary Figures


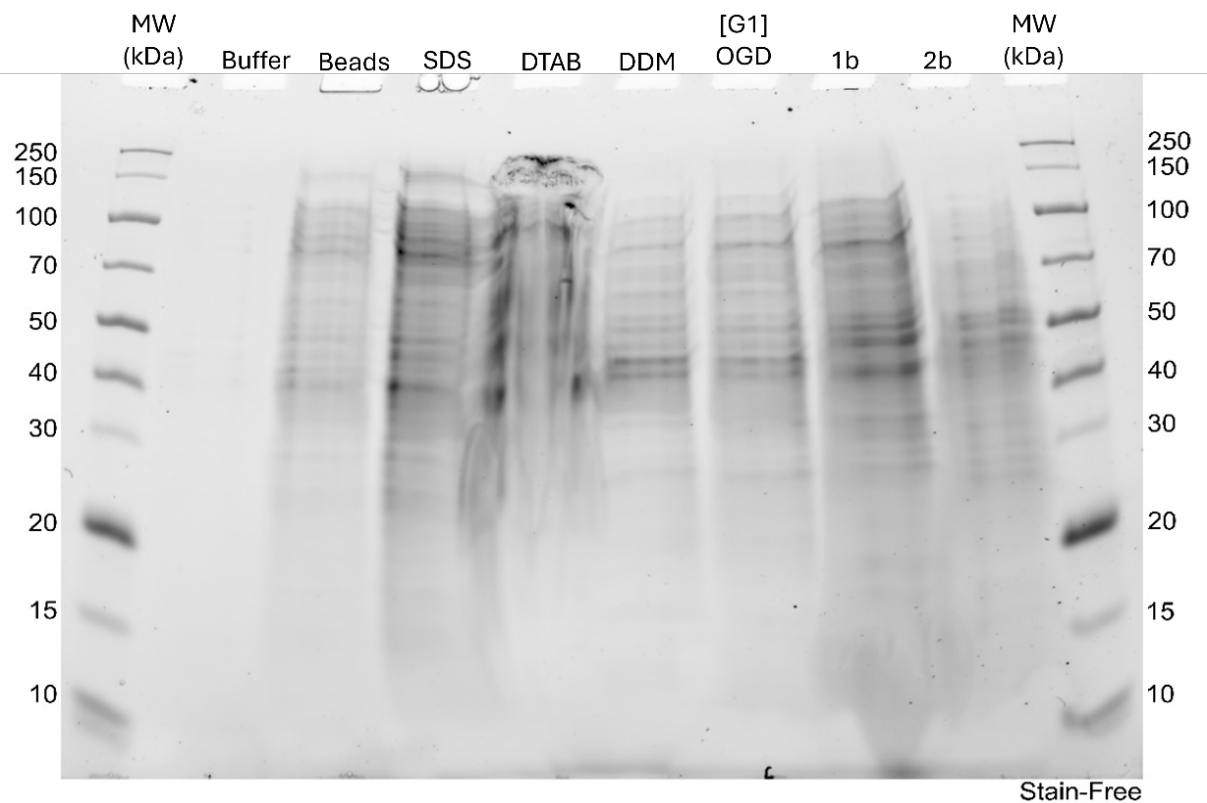


Fig. S1: SDS Page of protein samples from lysis using different detergents. The bands are named after the used lysis method, the ladder is marked as MW. (SDS=sodium dodecyl sulfate, DTAB=dodecyltrimethylammonium bromide, [G1] OGD= dendritic triglycerol detergent, DDM=Dodecyl-β-D-maltoside.)


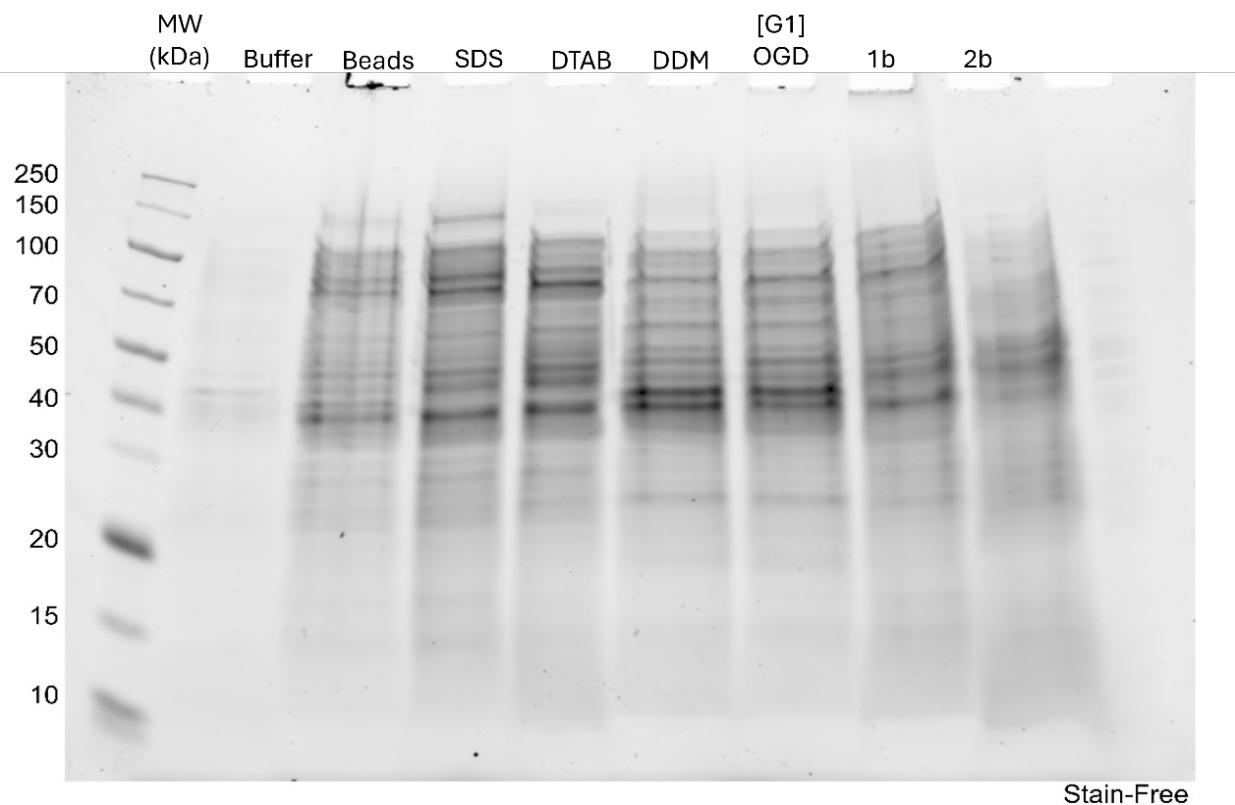


Fig. S2: SDS Page of protein samples from lysis using different detergents after acetone precipitation. The bands are named after the used lysis method, the ladder is marked as MW. (SDS=sodium dodecyl sulfate, DTAB=dodecyltrimethylammonium bromide, [G1] OGD= dendritic triglycerol detergent, DDM=Dodecyl-β-D-maltoside.)


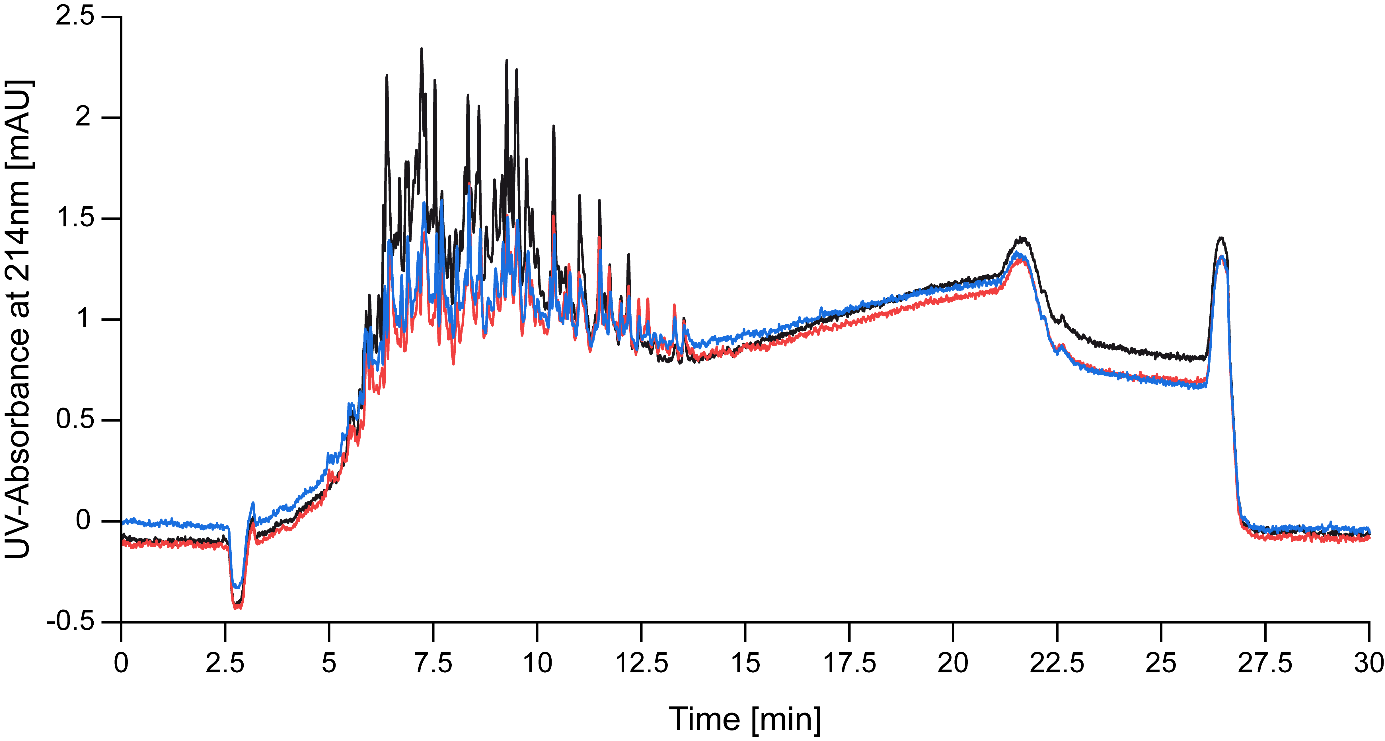


Fig. S3: SDS sample quality control chromatogram of peptide sample after on-filter digest. The graph shows UV-Absorbance at 214 nm over the retention time of three biological repeats in black, red and blue. (SDS=sodium dodecyl sulfate)


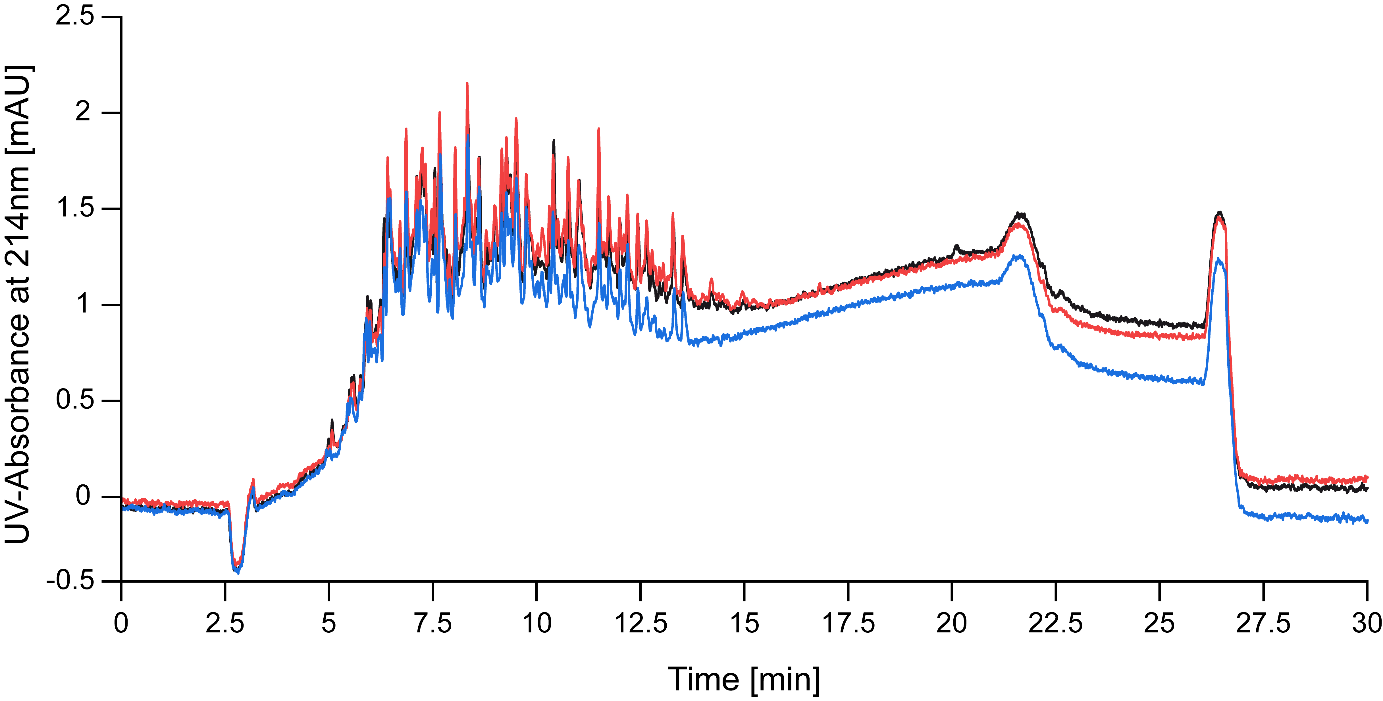


Fig. S4: DTAB sample quality control chromatogram of peptide sample after on-filter digest. The graph shows UV-Absorbance at 214 nm over the retention time of three biological repeats in black, red and blue. (DTAB=dodecyltrimethylammonium bromide)


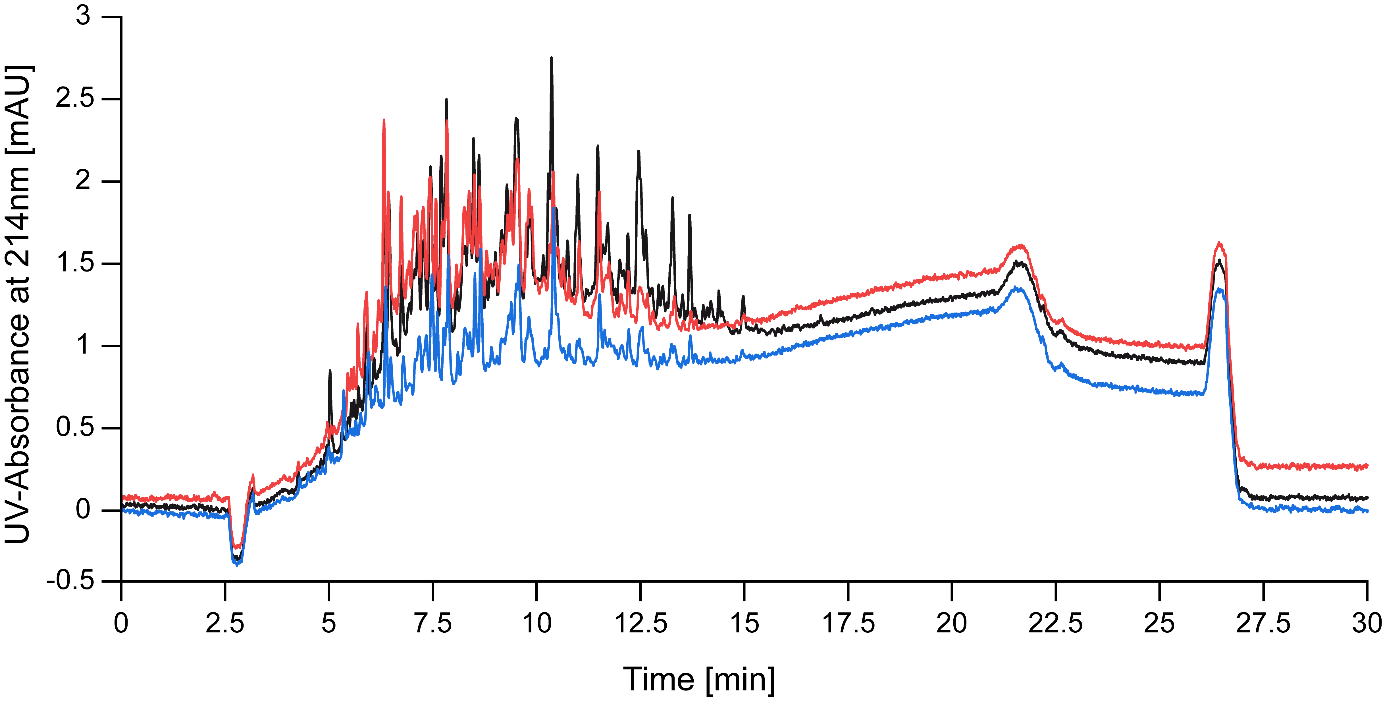


Fig. S5: [G1] OGD sample quality control chromatogram of peptide sample after on-filter digest. The graph shows UV-Absorbance at 214 nm over the retention time of three biological repeats in black, red and blue. ([G1] OGD= dendritic triglycerol detergent)


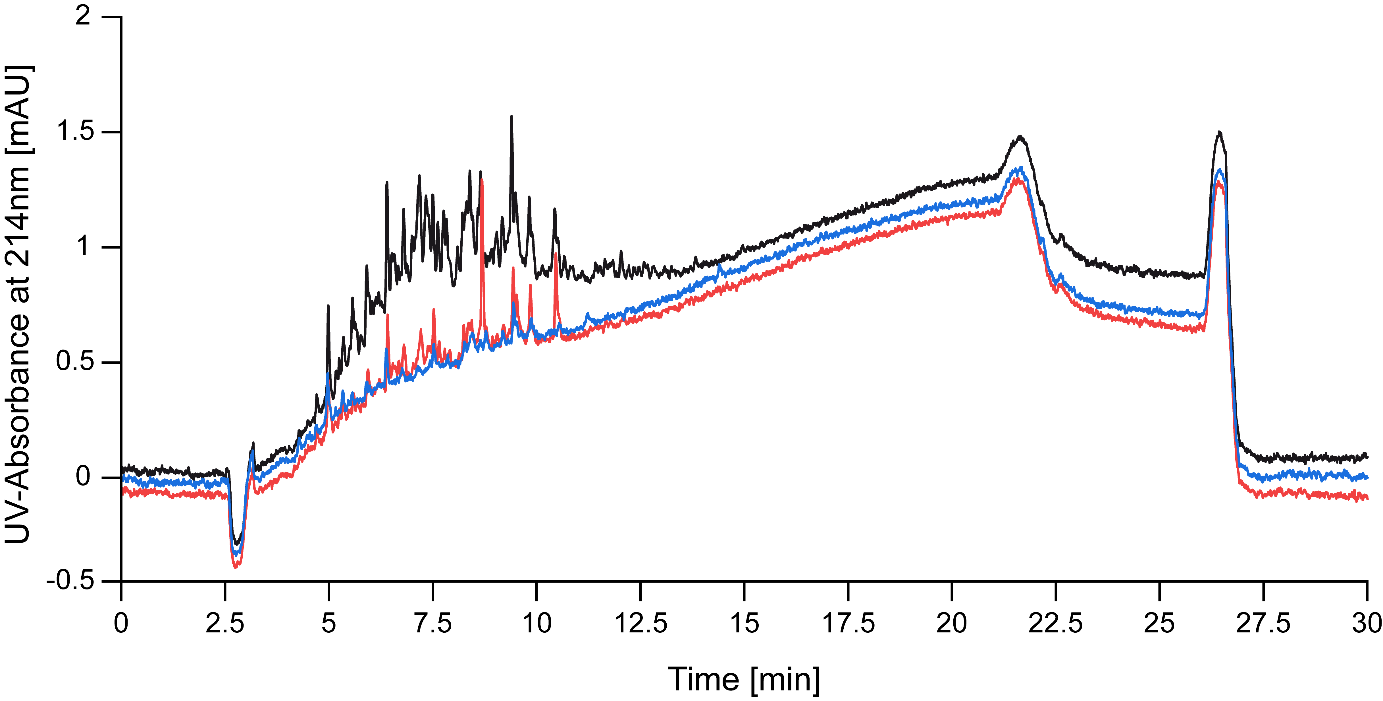


Fig. S6: **1a** sample quality control chromatogram of peptide sample after on-filter digest. The graph shows UV-Absorbance at 214 nm over the retention time of three biological repeats in black, red and blue.


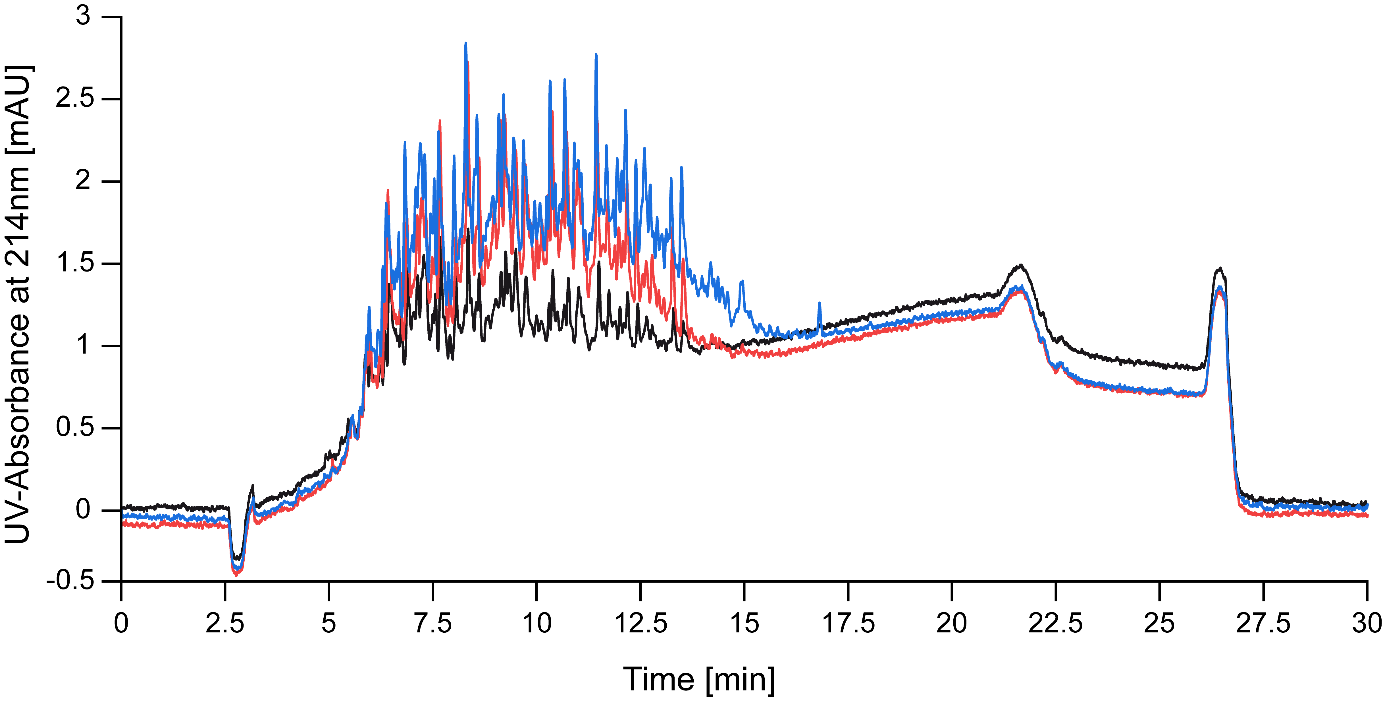


Fig. S7: **1b** sample quality control chromatogram of peptide sample after on-filter digest. The graph shows UV-Absorbance at 214 nm over the retention time of three biological repeats in black, red and blue.


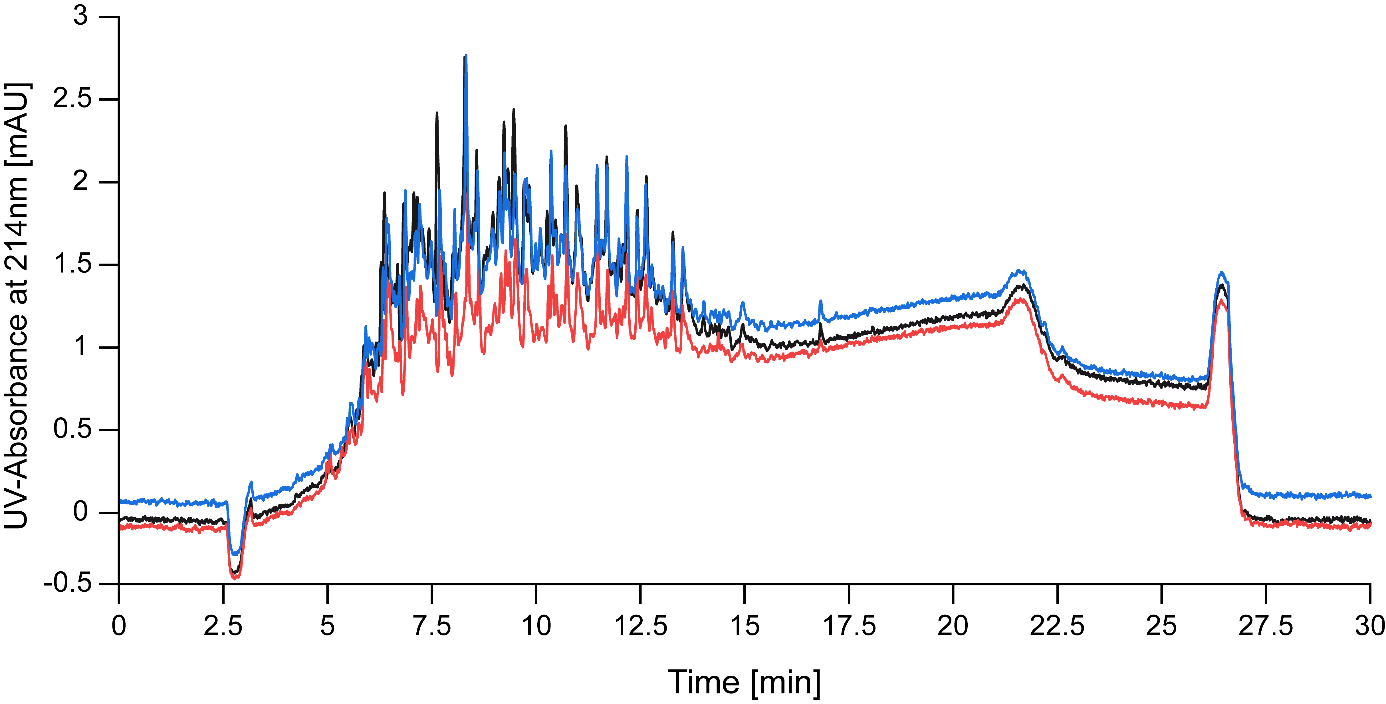


Fig. S8: **2b** sample quality control chromatogram of peptide sample after on-filter digest. The graph shows UV-Absorbance at 214 nm over the retention time of three biological repeats in black, red and blue.

# 2. Supplementary Tables

Tab. S1: Critical micelle concentration (cmc) of the detergents used in the present study (SDS=sodium dodecyl sulfate, DTAB=dodecyltrimethylammonium bromide, [G1] OGD= dendritic triglycerol detergent). Data was obtained from Wycisk *et al.*, 2024 (see reference below).

| **detergent** | **SDS** | **DTAB** | **[G1] OGD** | **1a** | **1b** | **2b** |
| --- | --- | --- | --- | --- | --- | --- |
| **cmc [mmol]** | 8 | 15 | 0.8 | 11 | 5.7 | 4.5 |

Wycisk, V., Behnke, J.-S., Nielinger, L., Seewald, M., Weisner, J., Binsch, M., Wagner, M.-C., Raisch, T., & Urner, L. H. (2024). Synthesis of Asymmetric Ionic Hybrid Detergents enables Micelles with Scalable Properties including Cell Compatibility. *Chem. Eur. J.*, *30*, e202401833.
